# Spontaneous Hepatic Rupture in a Pregnant Woman with Preeclampsia and HELLP Syndrome

**DOI:** 10.1155/2023/6683645

**Published:** 2023-04-03

**Authors:** Nguyen Phuoc Lam, Anh Tuan Mai, Thanh Chi Pham, Hieu Trung Kieu, Hien Quang Nguyen

**Affiliations:** ^1^Intensive Care Unit, Cho Ray Hospital, Ho Chi Minh City, Vietnam; ^2^University of Medicine and Pharmacy at Ho Chi Minh City, Vietnam

## Abstract

Spontaneous hepatic rupture is a rare complication associated with preeclampsia and is characterized by hemolysis, elevated liver enzymes, and a low platelet count (HELLP syndrome), with a nonspecific clinical presentation and high mortality rate. We present the case of a 34-year-old primigravida woman in whom spontaneous hepatic rupture associated with HELLP syndrome was accidentally detected during cesarean delivery. The patient was successfully managed with liver packing and transcatheter arterial embolization, followed by plasmapheresis. Spontaneous hepatic rupture should be considered in any HELLP syndrome patient presenting with epigastric or right upper quadrant pain and early signs of hemodynamic instability. A multimodal approach can help achieve good clinical outcomes in patients with this rare presentation.

## 1. Introduction

Hepatic rupture during pregnancy was first described by Abercrombie in 1844 [1]. It is a rare complication associated with preeclampsia and HELLP (hemolysis, elevated liver enzymes, and low platelet count) syndrome and can be life-threatening to both the mother and fetus. Spontaneous hepatic rupture occurs between 1/45,000 and 1/225,000 pregnancies; it has been reported in less than 2% of patients with HELLP syndrome [2]. Although the majority of hepatic ruptures occur in the late second or third trimester, one-third of the cases are reported in the postpartum period (often within 48 hours of delivery) [2–4].

Common clinical findings occur in 30–90% of the patients, including right upper quadrant (RUQ) pain (83.3%), hemodynamic instability (62.4%), nausea/vomiting (24.5%), right shoulder pain (13.2%), and abdominal distention (4.7%) [5–7]. Nevertheless, these signs and symptoms are nonspecific and may suggest the presence of other gastrointestinal issues, making the diagnosis of spontaneous hepatic rupture difficult [7]. Almost half of the patients had at least one symptom mentioned above, and 12.9% of patients were asymptomatic [7]. While a single sign or symptom is unspecific, the combination of these presentations (e.g., RUQ pain and shoulder pain) suggests a more severe etiology [8, 9]. A definite diagnosis of spontaneous hepatic rupture can be confirmed by transabdominal ultrasound or computed tomography; however, in some cases, hepatic involvement can only be identified during cesarean delivery due to maternal distress or nonreassuring fetal status [2]. Although mortality has been reduced over the past decades, the most recent largest systemic review showed that maternal and fetal mortality rates were 22% and 41.9%, respectively, with hemodynamic instability and preeclampsia/eclampsia being the independent predictors [7].

There has been a drastic improvement in hepatic rupture management over the last decades, with a shift toward endovascular treatment using transcatheter arterial embolization (TAE). Surgical control of bleeding in hepatic rupture can be challenging primarily because of the associated coagulopathy and also because infarctions and hematomas can be multifocal. As such, endovascular management is increasingly becoming the treatment of choice [10].

We report the case of a 34-year-old primigravida woman in whom spontaneous hepatic rupture associated with HELLP syndrome was incidentally detected during cesarean delivery. Treatment with hepatic packing, TAE, and therapeutic plasma exchange led to good clinical outcomes.

## 2. Case Presentation

A 34-year-old primigravida woman at 32 weeks of gestation without a history of diabetes mellitus, preeclampsia, or recent trauma was admitted to a local hospital for abdominal pain and transferred to a higher level care hospital. She complained of sudden and worsening RUQ pain, blurry vision, dizziness, and diaphoresis, which started three hours earlier. Her heart rate and blood pressure on admission were 125 bpm and 175/100 mmHg, respectively.

**Figure 1:**
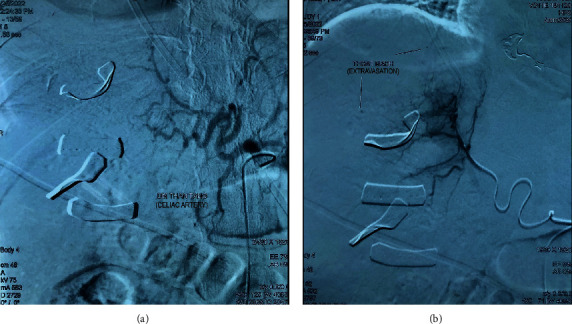
Extravasation signs on DSA. (a) TAE was performed via the celiac artery and (b) spot signs showing extravasation contrast. DSA: digital subtraction angiography; TAE: transcatheter arterial embolization. In the ICU, the patient was managed with mechanical ventilation, broad-spectrum antibiotics, fluid and blood transfusions, vasopressors, tranexamic acid, and magnesium sulfate for eclampsia prophylaxis.

A diagnosis of fetal distress complicated by severe preeclampsia and HELLP syndrome was made, and emergency cesarean delivery was performed. When the peritoneum was opened, approximately 300 g of blood was collected and suctioned. The uterus was unaffected, and a male newborn weighing 1590 grams was delivered. The Apgar scores were 5 and 6 at 1 and 5 min, respectively. Persistent bleeding from the liver fossa was suspected. The patient was infused with 10 units of packed red blood cells, 8 units of fresh frozen plasma, 8 units of cryoprecipitate, and 2 units of platelets, and a hepatobiliary surgeon was consulted. Bleeding from the right liver and rupture of Glisson's capsule in hepatic lobules V, VII, and VIII were identified. A diagnosis of HELLP syndrome complicated by a nontraumatic hepatic rupture was established. Pressure compression and liver packing were performed against the diaphragmatic surface at segments V, VII, and VIII, along with subhepatic drainage. Bleeding was controlled, and the patient achieved targeted blood pressure with vasopressors. The estimated blood loss was 2500 mL. Three hours after surgery, the patient was transferred to our hospital for hemodynamic instability. The heart rate and blood pressure while receiving norepinephrine infusion of 0.2 *μ*g/kg/min were 130 bpm and 110/70 mmHg, respectively. Six hours postoperatively, the patient's hemodynamic status rapidly deteriorated despite surgical packing; therefore, an emergency endovascular intervention was performed. Hepatic angiography revealed contrast media extravasation at many sites of the right hepatic artery; therefore, transcatheter arterial embolization with polyvinyl alcohol (PVA) and a gelatin sponge was performed (Figure 1). The patient was then admitted to the intensive care unit (ICU).

**Figure 2:**
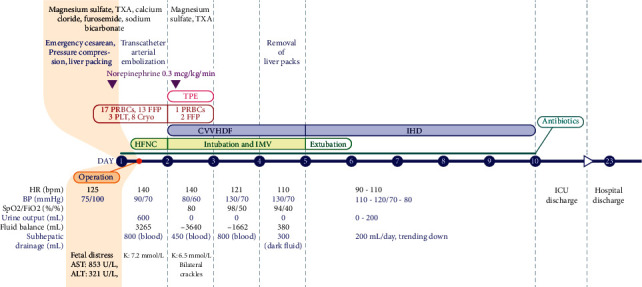
Progress of disease course. HR: heart rate; BP: blood pressure; SpO2: saturation of pulse oxygen; FiO2: fraction of inspired oxygen; PRBC: packed red blood cell; FFP: fresh frozen plasma; PLT: platelet; Cryo: cryoprecipitate; TPE: therapeutic plasma exchange; CVVHDF: continuous venovenous hemodiafiltration; IHD: intermittent hemodialysis; HFNC: high-flow nasal cannula; IMV: invasive mechanical ventilation; ICU: intensive care unit; bpm: beats per minute.

**Figure 3:**
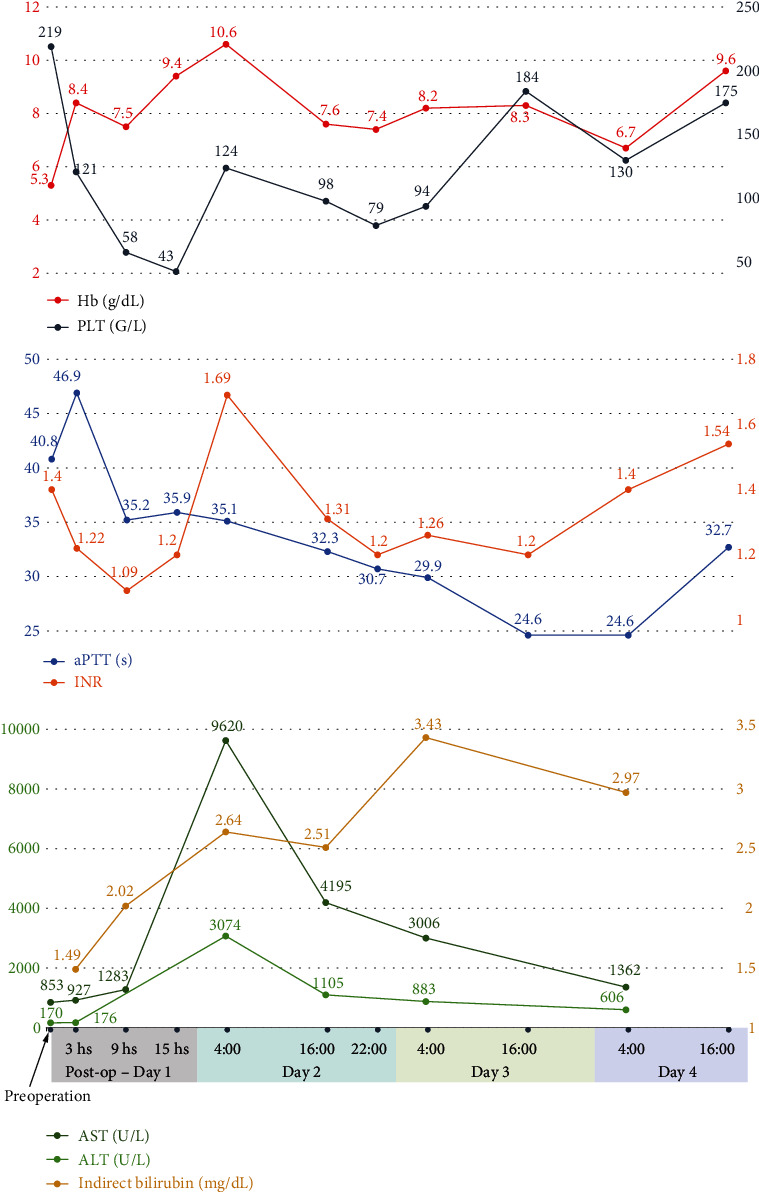
Variation of serum laboratory results over time.

On day 2 of ICU admission, thrombotic thrombocytopenic purpura (TTP) was suspected due to the constellation of anemia, thrombocytopenia, and hemolysis, as evidenced by elevated lactate dehydrogenase (LDH) and indirect bilirubin levels. Blood samples were collected for ADAMTS13 investigation, and 3-hour-long therapeutic plasma exchange (TPE) was performed, considering the moderate risk of TTP estimated by the PLASMIC score (5 points) [11]. Due to persistent hyperkalemia (6.5–7 mmol/L) nonresponsive to medication and anuria, continuous venovenous hemodiafiltration was performed after TPE (blood flow = 180 mL/min, filtration flow = 800 mL/h, dialysate flow = 1200 mL/h, and fluid removal flow = 50 mL/h). This was followed by three sessions of intermittent hemodialysis (blood flow, 200 mL/min; dialysate flow, 400 mL/min; and fluid removal flow = 2000 mL/4 h) due to oliguria. The patient's coagulation profile gradually improved, and the amount of drainage fluid decreased after TPE. The liver packs were removed on day 4 of ICU admission. The patient achieved stabilization and was extubated the following day. The patient was discharged from the ICU on day 10 and was managed in the hepatobiliary department with antibiotics and surgical wound care. The patient was discharged on day 23. The course of the disease and the variations in laboratory results are summarized in Figures 2 and 3.

## 3. Investigation

**Figure 4:**
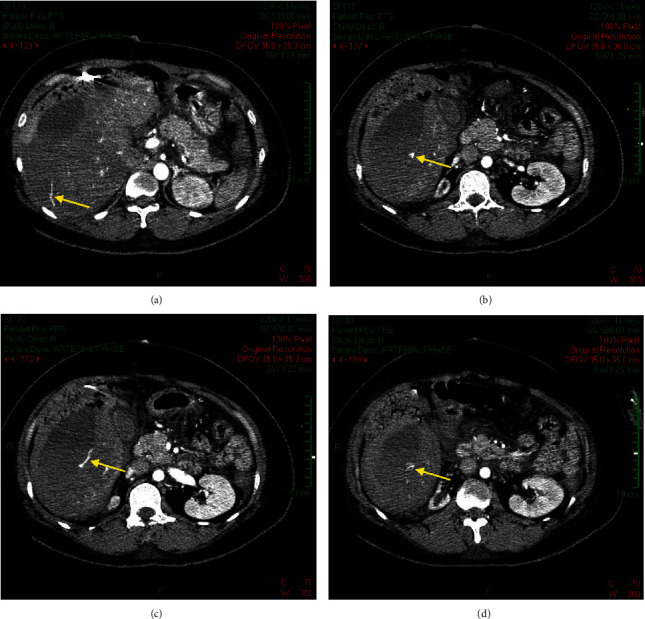
Subhepatic hematoma and subscapular active bleeding on CT scan at ED. (a, c) Active bleeding signs (yellow arrow) and (b, d) other extravascular contrast (yellow arrow). CT: computed tomography; ED: emergency department.

Serum laboratory results on initial admission were as follows: hemoglobin, 52.9 g/L; platelet count, 219 G/L; international normalized ratio (INR), 1.4; activated partial thromboplastin (aPTT), 40.8 seconds; fibrinogen level, 2.22 g/dL; aspartate aminotransferase (AST) level, 853 U/L; and alanine aminotransferase (ALT), 321 U/L. These results were consistent with the diagnosis of severe HELLP syndrome. In the emergency room of our hospital, the hemoglobin fluctuated at a low level, and the platelet count kept decreasing (Figure 3). An emergency abdominal CT scan revealed moderate intraperitoneal fluid, a subhepatic hematoma measuring 6 × 12 cm, with the packing, and signs of subscapular active bleeding (Figure 4). Arterial blood gas analysis showed severe metabolic acidosis (pH of 7.18 (7.35–7.45), PaCO_2_ of 29.2 (35–45) mmHg, and HCO_3_^−^ of 10.9 (22–26) mmol/L). Serum creatinine was 1.75 mg/dL (0.7–1.5), with an estimated glomerular filtration rate of 37.34 mL/min/1.73 m (normal > 60). Serum lactate was elevated at 18.08 mmol/L (0.5–2.2). Both direct and indirect Coombs' tests were negative. The investigations confirmed organ damage secondary to severe hemorrhagic shock from hepatic rupture.

**Figure 5:**
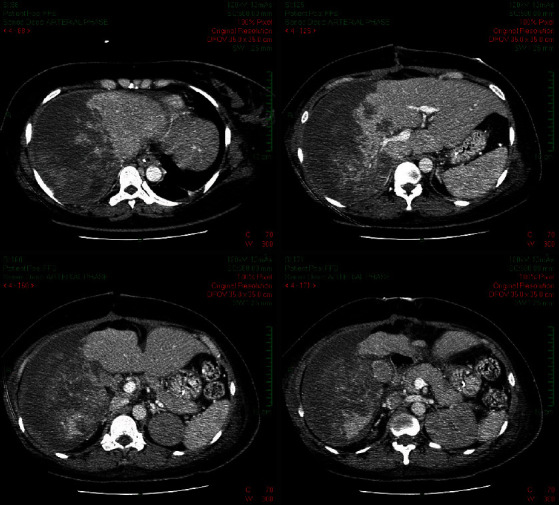
Perihepatic hematoma without signs of active bleeding on follow-up computed tomography.

The ADAMTS13 test obtained at ICU admission returned normal at 0.69 IU/mL (0.4–1.3), ruling out the diagnosis of thrombotic thrombocytopenic purpura (TTP). A follow-up abdominal CT scan obtained on ICU discharge showed a right perihepatic hematoma without signs of active bleeding in the liver (Figure 5). Urine analysis showed 3+ protein and 200 RBC/*μ*L on the same day. Other investigations were unremarkable.

## 4. Discussion

Although rare, spontaneous hepatic rupture is life-threatening; thus, clinicians should be highly suspicious when suggestive findings are observed in pregnant women with preeclampsia or HELLP syndrome. Most patients are accidentally diagnosed during cesarean delivery due to maternal or fetal distress, as in our patient's case [12]. As HELLP syndrome can occur in 83.7% of pregnant women with spontaneous hepatic rupture, emergency imaging (ultrasound, computed tomography scan, and magnetic resonance imaging) should be performed in these patients with unexplained abdominal pain, especially when compounded by the early signs of hypovolemic shock (e.g., tachycardia) [7, 13]. The pathophysiology is not fully understood. The suggested mechanisms of capsular rupture include periportal hemorrhage and intravascular fibrin deposition, resulting in sinusoidal obstruction, intrahepatic vascular congestion, and hepatic ischemia/necrosis [7].

Hemodynamic status is the determining factor for the management of preeclampsia complicating spontaneous hepatic rupture, and surgery is recommended if there is any evidence of hemodynamic instability [14]. There are two reported approaches for spontaneous hepatic rupture management in this population: conservative therapy, including embolization of the bleeding hepatic arteries and support with fluid and blood transfusion, and invasive therapy, including exploratory surgery, liver packing, resection, liver transplantation, or a combination of these techniques. Brito et al. reported 93 cases of hepatic rupture associated with gestational hypertension in 2021 [12]. Although most patients required surgical therapy for bleeding control, a few were successfully managed with conservative therapy and supportive care. In our patient's case, intra-abdominal bleeding was identified during cesarean delivery; therefore, exploratory surgery and liver packing were performed immediately. Unfortunately, the bleeding sites were poorly controlled, as extravasation of contrast media was detected on abdominal CT at our hospital. We performed minimal endovascular intervention. TAE is a recommended intervention for traumatic hepatic rupture and liver cancer; however, its application in spontaneous hepatic rupture associated with HELLP syndrome has been reported as minimal management in selected cases [6]. TAE has been reported to be a more effective and less invasive method for the management of hepatic rupture and postpartum disseminated intravascular coagulation than hepatectomy [7, 15]. We believe that after surgical liver packing, TAE could be considered as an alternative to liver resection surgery for spontaneous hepatic rupture associated with HELLP syndrome. Hepatectomy is the intervention of choice when the bleeding sites are not controlled by TAE.

There are numerous causes of thrombocytopenia in patients with HELLP syndromes, including sepsis, disseminated intravascular coagulation, thrombotic microangiopathies, and TTP, and the clinical presentation and laboratory values can be very confusing [16]. In our patient, a trial-plasmapheresis session was indicated for several reasons. First, a constellation of sudden thrombocytopenia, elevated LDH levels, anemia, and renal failure suggested TTP. We used the PLASMIC score to predict the probability of severe ADAMTS13 deficiency owing to the long turnover time of the ADAMTS 13 activity assay. Moderate risk (5–25%) of TTP was established by a score of 5 [11]. Second, the indication of TPE for postpartum HELLP syndrome can be individualized, as it falls in category III according to the most recent guidelines of the American Society for Apheresis [17]. Our patient was classified as having class I (platelet count < 50 G/L, AST/ALT ≥ 70 U/L, LDH > 600 U/L) HELLP syndrome with extremely high maternal mortality. TPE is an effective treatment modality for HELLP syndrome, particularly in those whose thrombocytopenia is nonresponsive to conservative therapy, as in the case of our patient [18]. In these patients, TPE helps remove several harmful serum components, including antibodies, immune complexes, endotoxins, and exotoxins, and replace serum proteins and coagulation factors [19]. Fresh frozen plasma was used as a replacement fluid for TPE. The optimal time for TPE initiation in HELLP syndrome is a matter of debate [20, 21]. Because of the overlap in clinical presentation and laboratory parameters between HELLP syndrome and thrombotic microangiopathies, we instituted TPE earlier than the time suggested by Martin et al. [20]; however, TPE performed within 48 h postpartum in the management of HELLP syndrome has been demonstrated to significantly improve mortality (*p* = 0.006) [22]. Third, maintaining coagulation hemostasis during hepatic rupture is crucial for bleeding control and may save patients from undergoing more invasive procedures, such as hepatectomy. While blood transfusion is a traditional approach to correct coagulation abnormalities, massive transfusion is associated with complications, including transfusion-related acute lung injury and pulmonary edema. Given her oliguric renal failure, the latter was most likely to occur in our patient. In this setting, TPE can be an alternative to correct coagulation abnormalities, control bleeding, and hasten recovery. Complete blood counts and coagulation parameters should be followed over consecutive days to assess the response to plasmapheresis. Those with a positive response to one session of trial-plasmapheresis can be considered suitable for other sessions; however, this approach lacks scientific evidence. In our patient, the ADAMTS13 levels recovered 2 days later, making severe HELLP syndrome complicated by spontaneous hepatic rupture the most likely diagnosis. Coagulation abnormalities had resolved after one session of TPE, and supportive care led to good clinical outcomes.

## 5. Conclusion

Spontaneous hepatic rupture should be considered in pregnant women presenting with sudden epigastric and/or RUQ pain accompanied by early signs of hemodynamic instability. In selected patients with hemodynamically unstable spontaneous hepatic rupture associated with HELLP syndrome, TAE can serve as an alternative to surgery to control bleeding. A session of trial-plasmapheresis may be performed early in the postpartum period if there are delays in the resolution of the HELLP syndrome and clinical findings suggestive of TTP.
